# A systematic review to assess the effectiveness of technology-based interventions to address obesity in children

**DOI:** 10.1186/s12887-020-02081-1

**Published:** 2020-05-22

**Authors:** Megan McMullan, Rachel Millar, Jayne V. Woodside

**Affiliations:** 1 School of Medicine, Dentistry and Biomedical Sciences, Queen’s University Belfast, University Road, Belfast, BT7 1NN UK; 2 Institute for Global Food Security (Centre for Public Health), Grosvenor Road, Belfast, BT12 6BJ UK

**Keywords:** Technology, Intervention, Obesity, Children, Web-based, Active video game, Exer-gaming, Internet

## Abstract

**Background:**

Childhood obesity is associated with a multitude of co-morbidities, including hypertension, hyperlipidaemia, cardiovascular disease and type 2 diabetes. Childhood obesity can also affect a young person’s social, emotional and mental health if they encounter negative prejudice and social marginalisation. Given the prevalence of overweight and obese children globally, it is imperative that effective interventions are developed. Children are receptive to information conveyed via digital means, therefore, the use of technology may play a crucial role in interventions to reduce childhood obesity. This systematic review aimed to review and critically appraise the literature published to date in relation to the effectiveness of technology-based interventions, employed as secondary prevention, in addressing childhood obesity.

**Methods:**

An electronic search strategy was undertaken in Medline and Embase, covering publications up to and including 12th July 2018. Randomised controlled trials assessing the effectiveness of technology-based interventions on weight-related outcomes in children, aged 8 to 18, published only in the English language, were included.

**Results:**

From an initial search total of 1012 studies, 11 met the inclusion criteria. They were assessed for methodological quality using the Cochrane Risk of Bias Tool for Randomised Controlled Trials and were analysed using a narrative approach. The findings of this review showed a limited potential of technology-based interventions, employed as secondary prevention, to address childhood obesity. Of the eleven studies reviewed, three (27%) showed a positive relationship between technology-based interventions and weight-related outcomes in overweight or obese children.

**Conclusions:**

This review suggests that technology-based interventions, primarily active video games, as well as internet or web-based interventions and mobile phone communications, may, with further research, have the potential to impact positively on weight-related outcomes. It is difficult to determine the degree of efficacy of these technology-based interventions, as only two databases were searched, selecting only English language articles. Moreover, the included studies demonstrated a lack of high-quality evidence. The lack and heterogeneity of studies with technology-based interventions is a further limitation.

## Background

Overweight and obesity are defined as conditions of abnormal or excessive body fat accumulation that exceeds healthy limits, presenting a risk to health [1]. Overweight and obesity is a worldwide epidemic among children and adolescents; in 2016 over 340 million children and adolescents aged 5–19 were overweight or obese [2]. Global age-standardised prevalence of obesity in girls demonstrated an increase from 0·7% in 1975 to 5·6% in 2016 and from 0·9% in 1975 to 7·8% in 2016 in boys [3]. Despite evidence of recent decline in childhood obesity rates in the USA [4], more than 12 million American children in the USA are obese — one in every six [5].

### Interpretation

The rising trends in children’s and adolescents’ BMI have plateaued in many high-income countries, albeit at high levels, but have accelerated in parts of Asia, with trends no longer correlated with those of adults.

Due to the prevalence of overweight and obese children globally, effective interventions are urgently required.

Childhood obesity is associated with a multitude of co-morbidities, including cardiovascular disease, type 2 diabetes and orthopaedic problems [6]. Childhood obesity may also affect the young person’s social and emotional health, as they can encounter negative prejudice and social marginalization [7]. This adverse stereotyping contributes to poor mental health, which can affect academic performance, resulting in restricted future employment opportunities [6].

Technology in the twenty-first century has become an integral part of everyday life for children. They live in a media-saturated environment, with 94% of 8 to 11 year olds and 99% of 12 to 15 year olds in the UK using a variety of technological devices [8]. The ensuing sedentary lifestyle can perhaps best be addressed by using the screen, their main source of entertainment and communication, as a medium to educate and motivate the child to adopt a healthier lifestyle.

Non-technological weight management interventions are not always accessible to children due to prohibitive cost, transport difficulties and lack of provision [9]. With up to 80% of children between 12 and 17 reluctant to engage in any form of organized sporting activity, there is a need for other innovative interventions [10].

To date, technology-based interventions, such as internet- and social media-based weight management programmes, smartphone apps and active video games have been developed to educate overweight and obese children [11–13]. These interventions are readily accessible, inexpensive and time-saving, allowing the child to engage independently from home with anonymity.

An et al. [14] conducted a systematic review of randomised controlled trials (RCTs) examining the effect of web-based weight management programs for children and adolescents. The authors found that six of eight studies confirmed that internet-based interventions, either alone or in combination with other behavioural interventions, had significant beneficial effects on reported outcomes. Two of three studies that compared internet-based interventions to a non-intervention group established that the BMI in internet-based intervention groups was significantly reduced. The time scale of these effective interventions varied, all studies covered a minimum period of twelve weeks and two continued for 2 years. The review focused on internet-based interventions only, rather than the full range of technology-based interventions employed as secondary prevention to address childhood obesity.

As children spend a significant amount of time on their technological devices, playing active video games or exergaming allows them to become more active and expend more energy [15]. Exergaming is using video games as a form of exercise. A systematic review has indicated that exergames generate moderate intensity levels of physical activity as well as being enjoyable. A 4 month RCT reported on the positive effects of active video games on weight and physical activity (PA) in a cohort of overweight and obese children [16].

To date, most research has focused on the effectiveness of technology-based interventions as primary prevention of childhood obesity. For example, Chen at al [17]. assessed the efficacy of technology-based interventions for obesity prevention in adolescents. There is a lack of knowledge on the impact of a full range of technology-based interventions on weight management in already overweight and obese children. The aim of this review was, therefore, to investigate the effectiveness of technology-based interventions, employed as secondary prevention, in addressing childhood obesity.

## Methods

This systematic review was conducted in accordance with the PRISMA (Preferred Reporting Items for Systematic Reviews and Meta-Analysis) guidelines [18]. There was no published review protocol. Eligibility criteria were:participants aged from 8 to 18 years, all or some of whom had to be overweight or obese. Three established definitions for overweight and obesity were accepted [19–21]. Technology-based interventions, such as social media, mobile phones, websites and active video games addressing childhood obesity, occurring in all settings and with the aim of decreasing BMI and other weight related outcomes, were included. Intervention period was minimum 2 weeks. The control was non-technology-based interventions or the stated technology-based interventions compared with each other. The primary outcome was BMI and secondary outcomes were other weight related outcomes, including weight, BMI percentile, BMI z-score, percentage body fat (% BF), waist circumference (WC) and waist to hip ratio (WHR). Only RCTs, including parallel group, cluster randomised and randomised cross-over trials, were allowed. Excluded were participants outside the specified age, those with a medical condition impacting on diet, weight or ability to exercise and studies comprising only of normal or underweight children. Studies examining technology-based intervention as part of a multicomponent intervention and studies published in a language other than English were excluded.

A search strategy was undertaken to identify relevant studies for inclusion. The search was last updated on 13th July 2018, in Medline and Embase.

Searches employed the AND combination of two main concepts: Obesity and Technology. Key words, including synonyms, closely related words and spelling variations, for each of the two concepts were combined using the operator ‘OR’. The searches were restricted to RCTs, English language and human only studies. Figure S1 demonstrates the search strategy for Embase. (See Additional file 1).

Duplicate studies were removed, as titles of studies considered irrelevant. Abstracts of potentially relevant titles were screened for inclusion, followed by full texts, and full texts were reviewed by a second reviewed for consensus (RM), with divergence resolved through discussion. Reference lists of included articles were also reviewed for eligibility.

The following data were extracted: authors and year of publication, study design, study location, number of participants in analysis, gender, age and characteristics of participants, study retention rate, the intervention and control, intervention duration and intensity, outcomes measured, follow up details and key findings.

Methodological quality was evaluated using the Cochrane Risk of Bias Tool for Randomised Controlled Trials [22], with results translated to the Agency for Healthcare Research and Quality (AHRQ) standard of ‘good’, ‘fair’, and ‘poor’ quality, using established conversion thresholds [22]. RevMan was used to produce a ‘Risk of bias table’, a ‘Risk of bias graph’ and a ‘Risk of bias summary’.

A narrative approach to data synthesis was used, rather than meta-analysis, primarily due to the heterogeneous nature of the included studies.

## Results

One thousand twelvearticles were obtained from Medline (*n* = 204) and Embase (*n* = 808) and were screened to remove duplicates. Titles and abstracts of the remaining 875 unique articles were screened and 851 studies rejected. Two reviewers independently examined the full text of the 24 remaining articles for inclusion. Eight articles were excluded due to participants being outside the specified age range; two others because they described primary prevention interventions; a further two due to insufficient participant information and one because the technology-based intervention was part of a multicomponent intervention. Therefore a total of 11 studies were considered suitable for inclusion. No further articles were retrieved from the bibliography search. Figure S2 demonstrates the study selection process (See Additional file 2).

An overview of study characteristics extracted for this review is presented in Table 1. Year of publication ranged from 2006 [23] to 2017 [24]. Studies were all RCTs, three of which were cluster RCTs, where two randomised schools [25, 26] and one randomised a mixture of schools and YMCAs [16]. The studies were carried out in a range of countries, with the majority taking place in the USA (*n* = 6) [9, 16, 23, 24, 27, 28] and others in New Zealand (*n* = 2) [29, 30], Canada (*n* = 1) [31], Malaysia (n = 1) [26] and the Netherlands (n = 1) [25]. Study retention rates ranged from 70.2% [23] to 100% [26, 30].

**Table 1:** Characteristics of Selected Studies

Authors & Year	Study Design	Country	Participants				Retention Rate (%)
			Number in Analysis	Gender	Age (years):	Characteristics	
			1) Intervention		Range or Mean (SD)		
			2) Control				
Adamo et al., 2010	RCT	Canada	1) 13	M + F	12–17	- Overweight or obese (CDC definition)	86.7
			2) 13				
Chen et al., 2011	RCT	USA	1) 26	M + F	12–15	- Normal weight, overweight or obese (CDC definition)	93
			2) 24		12.52 (3.15)	- Chinese-American	
Ezendam et al., 2012	Cluster RCT	Netherlands	1) 400 (11 schools)	M + F	12–13	- Students recruited from 20 schools	86
			2) 342 (9 schools)			- Analysis was conducted for the total study population and repeated for at risk students (those not meeting behavioural recommendations at baseline)	
						- At risk students were normal, overweight or obese (IOTF definition)	
Jones et al., 2008	RCT	USA	1) 44	M + F	15.1 (1.0)	- High school students	82.3
						- Overweight or obese (CDC definition)	
			2) 43			- Binge eating or overeating behaviours	
						- African American, Hispanic or Other	
Maddison et al., 2011	RCT	New Zealand	1) 160	M + F	10–14	- Overweight or obese (IOTF definition)	100
			2) 162		11.6 (1.1)	- Play ≥2 h of video games per week	
						- Māori, Pacific Islanders, European or Other	
Nawi & Jamaludin., 2015	Cluster RCT	Malaysia	1) 47 (4 schools)	M + F	16	- Students with a BMI > 25 kg/m^2^	100
			2) 50 (2 schools)			- Malaysian or Non-Malaysian	
Nguyen et al., 2013	RCT	New Zealand	1) 78	M + F	13–16	- Overweight to moderately obese (BMI z-score: 1.0–2.5)	80
			2) 73				
						- African American, Caucasian or Hispanic	
Staiano et al., 2017	RCT	USA	1) 20	F	14–18	- Overweight or obese CDC definition)	92.7
			2) 18		16 (1.4)	- African American or Caucasian	
Trost et al., 2014	Cluster RCT	USA	1) 34 (4 YMCAs and 2 schools)	M + F	8–12	- Overweight or obese (CDC definition)	92
			2) 41 (3 YMCAs and 2 schools)		10 (1.7)	- African American or Caucasian, Hispanic, Asian or Mixed	
Wagener et al., 2012	RCT	USA	1) 20	M + F	12–18	- Obese (CDC definition)	98
			2) 20		14 (1.66)	- African American, Caucasian, Hispanic or Biracial	
Williamson et al., 2006	RCT	USA	1) 29	F	11–15	- African American girls with one overweight (BMI > 27) or obese (BMI > 30) biological parent	70.2
			2) 28		13.2 (1.4)	- Overweight or obese (CDC definition)	

Abbreviations – *SD* standard deviation, *RCT* randomised controlled trial, *USA* United States of America, M: male, *F* female, *CDC* Centres for Disease Control and Prevention, *IOFT* International Obesity Task Force, *BMI (kg/m*^*2*^*)* body mass index (kilograms/meters^2^)

The total number of participants analysed varied from 26 [31] to 742 [25], with an age range from 10 years [16] to 16 years [24]. The majority of studies included both genders, with a small number of studies conducted among females only (n = 2) [23, 24]. Two studies focused exclusively on participants of one particular ethnicity; one focused on Chinese-American adolescents [27], while the other focused on African-American girls [23]. Two studies did not specify ethnicity [25, 31], while the remaining seven studies focused on children from a variety of ethnic origins [9, 16, 24, 26, 28–30].

All studies focused on technology-based interventions as secondary prevention, since participants were overweight or obese prior to commencement of study. However, two studies also included participants who were normal weight and presented this data separately. In this case, the intervention also served as primary prevention [25, 27].

One study included participants who engaged in binge eating or overeating behaviours [9]. Two studies had a family component, one of which provided three 15-min internet sessions for the parents so that they might acquire the skills to support their children in leading a healthy lifestyle [27]. The other study required the adolescents to participate in the study with a parent [23].

There was some heterogeneity in how weight categories were defined. Seven studies used the CDC definition [9, 16, 23, 24, 27, 28, 31] and two studies used the IOTF definition to explain the terms normal, overweight and obese [25, 30]. Two studies did not employ an established definition; one required participants to have a BMI > 25 kg/m^2^ [26], while the other classed its participants as overweight or obese with a BMI z-score between 1.0 and 2.5 [29]

Details of the intervention and control groups, outcomes, follow-up time points and key findings, including the effectiveness of interventions on BMI and other weight-related outcomes, for each included study were noted. The mean difference of weight-related outcomes between intervention and control groups was also calculated.

Technology-based interventions were sub-divided into three main categories; active video games or exergaming, internet or web-based interventions and mobile phone communications. Intervention duration ranged from 8 weeks [27] to 2 years [23, 29]. Intervention intensity was reported in all but two studies [16, 26] and was highly heterogeneous. Follow-up time points varied across studies from between 2 months [27, 29] and 24 months [23, 25, 29]. Four studies compared a technology-based intervention to a nil intervention control group [9, 24, 25, 28]; four studies compared a technology-based intervention to a non-technology-based intervention, and these included stationary cycling to music [31], written pamphlets [26] and lifestyle programmes [16, 29]. Three studies compared the effectiveness between different technology-based interventions, however, the intervention of interest had an interactive or tailored component compared to the passive or sedentary technology-based control intervention [23, 27, 30]. The study outcomes varied depending on the aims of the studies but they all included a BMI outcome, given as either BMI, BMI z-score or BMI percentile. Further common outcomes were other anthropometric, dietary and PA measurements.

### Active video game interventions

Five studies explored the effectiveness of active video gaming or exergaming [16, 24, 28, 30, 31] (Table 2).

**Table 2:** Intervention and Control Characteristics, Outcomes, Follow-up Time Points and Key Findings for Studies Investigating the Effects of Active Video Games

Authors & year	Interventions	Intervention intensity & duration	All outcomes	Follow-up time points	Key findings	Weight-related outcomes	Between group mean difference	*P*-value*
							(95% CI)	
Adamo et al., 2010	1) GameBike - interactive video game and stationary cycling 2) Stationary cycling to music	Twice weekly 60-min sessions for 10 weeks	1) Exercise adherence and behaviour 2) Aerobic fitness 3) Body composition 4) Metabolic profile 5) Diet	Post treatment	- The music group attended a statistically significantly higher percentage of sessions compared to the video game group (92 vs. 86, *p* < 0.05) - Minutes spent at vigorous intensity (24.9 ± 20 vs. 13.7 ± 12.8, *p* < 0.05) and average distance (km) pedalled per session (12.5 ± 2.8 vs. 10.2 ± 2.2, *p* < 0.05) were statistically significantly greater in the music group - No difference in all other outcomes between groups - Both groups had a statistically significant reduction in peak HR, an improvement in peak workload and a reduction in time to exhaustion from pre to post intervention (*p* < 0.05)	BMI (kg/m2)	−3.9 (− 11.1, 3.3)	0.3
						BMI percentile	- 0.3 (− 1.89, 1.29)	0.74
						Body weight (kg)	−13.1 (− 30.52, 4.32)	0.15
						WC (cm)	−4.3 (− 16.46, 7.86)	0.53
						% BF	1.4 (−6.24, 9.04)	0.72
Maddison et al., 011	1) Active video games 2) Sedentary video games	Children were encouraged to play for 60 min most days of the week, for a period of 24 week	1)Weight 2) BMI 3) BMI z-score 4) % BF 5) WC 6)PA 7) Cardiorespiratory fitness 8) Video game play 9) Food snacking	12 and 24 weeks	At 24 weeks, statistically significantly more children in the intervention group than the control group had: - decreased their BMI (−0.24, *p* = 0.02), BMI z-score (− 0.06, *p* = 0.04), BF % (− 0.83, *p* = 0.02) and body weight (− 0.72, *p* = 0.02) - increased average daily time of active video game play (10.03, *p* = 0.0001) - no difference in all other outcomes between groups	BMI (kg/m^2^)	−0.24 (− 0.44, − 0.04)	**0.02**
						BMI z-score	0.06 (−0.12, − 0.03)	**0.03**
						Body weight (kg)	−0.72 (−1.33, − 0.10)	**0.02**
						WC (cm)	−1.21 (−2.45, 0.03)	0.22
						% BF	−0.83 (−1.54, − 0.12)	**0.02**
Staiano et al., 2017	1) Group-based dance exergaming 2) Self-directed care control condition	Three 1-h sessions per week for 12 weeks	1) Body composition: BMI percentile, BMI z-score, WC, %BF, regional BF, visceral and subcutaneous abdominal adiposity 2) Cardiovascular risk factors	Within 2.5 weeks post-intervention	- There were no statistically significant group differences in any body composition or cardiovascular risk factor outcome	BMI z-score	−0.01 (− 0.069, 0.049)	0.74
						BMI percentile	−0.02 (− 0/78, 0.39)	0.51
						WC (cm)	−0.04 (−3.37, 3.29)	0.98
						% BF	−0.3 (−1.28, 0.68)	0.55
Trost et al., 2014	1) Paediatric weight management programme plus active video gaming 2) Programme only	16 weeks Intensity not stated	1) Daily moderate-to-vigorous and vigorous PA 2) % overweight 3) BMI z-score	8 and 16 weeks	- At week 16, compared to the programme only group, the programme plus active video gaming group had statistically significant increases in moderate-to-vigorous (8.0, *p* = 0.04) and vigorous (3.1, *p* = 0.02) PA - At week 16, both groups had statistically significant reductions in % overweight and BMI z-score but the programme plus active gaming group had statistically significantly greater reductions (−10.9, *p* = 0.02),(−0.25, *p* < 0.001)	BMI z-score	−0.16 (− 0.19, − 0.13)	**< 0.001**
Wagener et al., 2012	1) Group dance-based exergaming programme 2) Wait-list control group	3 sessions a week for 10 weeks: 1st session: 40 min 2nd and 3rd session: 75 min each	1) BMI z-scores 2) Self-reported psychological adjustment and perceived competence to exercise 3) Maternal reported adolescent psychological adjustment	10 weeks	- Compared with control group, participants in the dance-based exergaming group statistically significantly increased in self-reported perceived competence to exercise - Maternal report of adolescent externalizing and internalizing symptomatology also decreased from baseline to end-of-treatment - No differences in BMI z-score within or between conditions	BMI-z score	0.01 (−0.12, 0.13)	0.87

*Abbreviations* - *BMI (kg/m2)* body mass index (kilograms/meters2), *WC (cm)* waist circumference (centimetres), *% BF* percentage body fat, *WHtR* waist-to-hip ratio, *HR* heart rate, *PA* physical activity

*P-values in bold are statistically significant at 5% significance level

Adamo et al. [31] investigated the effectiveness of an interactive video game which involved stationary cycling, known as ‘GameBike’, compared to stationary cycling to music. The study focused on exercise adherence, energy expenditure, aerobic fitness, body composition, and cardiovascular disease risk markers in overweight and obese adolescents. The author found that the music group had significantly better adherence and expended more energy than the ‘GameBike’ group. There were no other notable differences between the two groups. Within both groups, statistically significant findings included a reduction in peak heart rate (HR) at peak workload, an improvement in peak workload and a reduction in time to exhaustion from pre to post intervention. There were no statistically significant between or within group differences for BMI.

Furthermore, in the second study, Maddison et al. [30] explored the effect of active video games on weight, body composition, PA and physical fitness compared to the effect of sedentary video games. This study established that children in the active video games group had significantly decreased their BMI, BMI z-score, % BF and body weight compared to the children in the sedentary video games group.

The objective of the third study by Staiano et al. [24] was to examine the effect of exergaming on adolescent girls’ body composition and their cardiovascular risk factors compared to a control group who adhered to their normal PA level. No statistically significant differences in body composition or cardiovascular risk factors between the two groups were found at follow-up.

The aim of the fourth study by Trost et al. [16] was to evaluate the effects of active video gaming on PA and weight loss in children participating in a community-based weight management programme. Statistically significant increases in PA were confirmed in the active video gaming group compared to the control group. The control group participated only in the programme and had no access to active video gaming. Both groups had statistically significant reductions in percentage of children overweight and BMI z-scores, however, the active gaming group demonstrated statistically significantly greater reductions.

Finally, Wagner et al. [28] investigated the impact of dance-based exergaming on obese adolescents’ perceived ability to exercise, their psychological adjustment and their BMI compared to a wait-list control group. It was noted that there was a statistically significant increase in self-reported perceived ability to exercise compared to the control group. However, no statistically significant differences were found in BMI z-score within or between the two groups.

In conclusion, two out of these five studies demonstrated a statistically significant mean difference for the intervention compared to the control for all or some of their weight-related outcomes; Maddison et al. [30] found beneficial results for four weight-related outcomes. BMI, BMI z score, body weight and % BF, with a statistically significant mean difference of − 0.24, − 0.06, − 0.72 and − 0.83 respectively when compared to control group, with *P*-values of 0.02, 0.03, 0.02 and 0.02. Trost et al. [16] found a statistically significant mean difference of − 0.16 for BMI z-score when compared to the control group with a P-value of < 0.001. The remaining findings were not statistically significant on between and within group analyses and displayed a degree of heterogeneity in estimates in terms of trend of direction.

### Internet-based interventions

Five studies examined the effect of web-based or internet interventions (Table 3) [9, 23, 25–27].

**Table 3:** Intervention and Control Characteristics, Outcomes, Follow-up Time Points and Key Findings for Studies Investigating the Effects of Internet-based Interventions

Authors & year	Interventions	Intervention intensity & duration	All outcomes	Follow-up time points	Key findings	Weight-related outcomes	Between group mean difference	*P*-value*
							(95% CI)	
Chen et al., 2011	1) Taored web-based intervention 2) General health, web-based information	Weekly online sessions for 8 weeks	1) BMI 2) WHtR 3) BP 4) Dietary intake 5) PA; knowledge and self-efficacy 6) Nutrition	2, 6, and 8 months	Statistically significantly more adolescents in the intervention group than the control group had: - decreased their WHtR (−0.01, *p* = 0.02) - decreased their DBP (− 1.12, *p* = 0.02) - increased PA as measured by the actigraph (12.46, *p* = 0.01) - increased FV intake (0.14, *p* = 0.001) - increased knowledge of PA (0.16, *p* = 0.008) and nutrition (0 .18, *p* = 0.001) - Statistically significant within group changes for the intervention group included WHtR, DBP, PA, FV intake and knowledge related to PA and nutrition (*p* < 0.05) -There were no statistically significant changes for any outcomes in the control group	BMI (kg/m^2^)	0.01 (−0.03, 0.04)	0.84
						WHtR	−0.01 (− 0.01, − 0.001)	**0.02**
						% BF	0.24 (− 0.49, 0.01)	0.06
Ezendam et al., 2012	1) FATaintPHAT- computer-tailored intervention 2) No intervention control group	15 min allocated for each of 8 lessons timetabled into regular school curriculum over 10 weeks	1) Self-reported behaviours (diet, PA, sedentary behaviour) 2) Pedometer counts 3) BMI 4) WC 5) Fitness	4 months and 2 years	Among the students at risk, those in the intervention group, compared to the control group: - reported eating more FV at 4-month follow-up (0.3, *p* = 0.02) - reported more steps at 2-year follow-up (12,389, *p* = 0.03) - The intervention had no effects on anthropometric outcomes or on sedentary behaviour	BMI (kg/m^2^)	0.25 (− 0.29, 0.79)	0.37
						WC (cm)	1.3 (−0.12, 2.72)	0.08
Jones et al., 2008	1) SB2-BED - an internet-based weight maintenance program 2) Wait-list control group	Over 16 weeks, a new topic was introduced weekly with previous content accessible at any time	1) BMI 2) Binge eating behaviours 3) Dietary fat and sugar intake 4) Depression 5) Programme adherence	Post treatment and 9 months	- Compared to the wait- list control group, the SB2-BED group had significantly reduced weight and shape concerns from baseline assessment to follow-up assessment at 9 months (−0.33, *p* = 0.03) - No difference in all other outcomes between groups - Statistically significant reductions in OBEs and SBEs from baseline assessment to posttreatment assessment (*p* < 0.01) and from baseline assessment to follow-up assessment (*p* < 0.05) were observed among SB2-BED participants	BMI (kg/m^2^)	−1.4 (−3.87, 1.05)	0.26
						BMI z-score	−0.16 (−0.41, 0.09)	0.21
Nawi & Jamaludin., 2015	1) ObeseGO! – healthy lifestyle website 2) Health education pamphlets	12 weeks Intensity not stated	1) BMI 2) WC 3) % BF	Post treatment and at 12 weeks post-intervention	- There was no statistically significant reduction in BMI, WC or BF % between the intervention and control groups - The mean BMI, WC and % BF in the obeseGO! group were statistically significantly lower after the intervention (*p* < 0.001, *p* = 0.001, *p* = 0.001)	BMI (kg/m^2^)	−0.49 (−2.41, 1.42)	0.61
						WC (cm)	−1.57 (−6.18, 3.03)	0.5
						% BF	0.09 (−2.14, 2.33)	0.93
Williamson et al., 2006	1) Interactive behavioural internet programme 2) Passive internet health education programme	Programmes continuously available for use over 24 months	1) BMI 2) BMI percentile 3) Body weight 4) % BF 5) Weight loss behaviours: dieting, weight concerns, exercise, overeating, and avoidance of fattening foods 6) Website use	6, 12, 18 and 24 months	- In comparison with the control group, adolescents in the behavioural programme statistically significantly reduced their % BF (−1.12 (0.47) vs 0.43 (0.47), p < 0.05) during the first 6 months. However, after 2 years, % BF did not differ between the two groups (−0.08 (0.71) vs. 0.84 (0.72), *p* > 0.05) - Adolescents in both the treatment and control groups reported improvement in exercise and overeating, in comparison with baseline (*p* < 0.05)	BMI (kg/m2)	- 0.47 (− 2.29, 1.35)	0.61
						% BF	−0.9 (−2.9, 1.06)	0.37
						BMI percentile	−0.003 (− 0.011, 0.0053)	0.48

*Abbreviations* - *BMI* body mass index, *WHtR* waist to height ratio, *BP* blood pressure, *PA* physical activity, *DBP* diastolic blood pressure, *FV* fruit and vegetables, *WC* waist circumference, *SB2-BED* Student Bodies 2 – BED, *OBEs* objective binge episodes, *SBEs* subjective binge episodes, *% BF* percentage body fat

*P-values in bold are statistically significant at 5% significance level

Chen et al. [27] aimed to examine the efficacy of a theory-driven, family-based online programme to promote healthy lifestyles and weights in Chinese-American adolescents. This was compared to guidance given on a general health website. Outcomes included BMI, Waist-to-hip ratio (WHtR), diet, PA and knowledge about PA and nutrition. Chen et al. [27] found that adolescents in the intervention group, compared to the control group, had statistically significantly decreased their WHR and their diastolic blood pressure (DBP) while statistically significantly increasing their PA, improving their diet and increasing their knowledge in regard to PA and nutrition. Statistically significant within group changes for the intervention group included WHtR, DBP, PA, fruit and vegetable intake and knowledge related to PA and nutrition. There were no statistically significant within-group changes for any outcomes within the control group.

Similarly, Ezendam et al. [25] conducted a study to evaluate the short- and long-term results of FATaintPHAT, a web-based computer-tailored intervention which aimed to increase PA, decrease sedentary behaviour and promote healthy eating in adolescents. The control group was a nil intervention group. Outcomes included self-reported behaviours in terms of diet, PA and sedentary behaviour, step count, fitness measured by a shuttle run test, as well as anthropometric measures. Analysis for this study was conducted for the total study population and repeated for all at risk students, namely those who did not meet behavioural recommendations at baseline. This study found that, for at risk students, the intervention had no statistically significant effect on anthropometric outcomes or on sedentary behaviour but did show a statistically significant increase in fruit and vegetable consumption at 4 month follow-up and increased steps at 2 year follow up. For the total study population, FATaintPHAT had no statistically significant effect on BMI, WC or percentage of overweight or obese students. However, at 4 month follow-up, the intervention group was less likely to report drinking more than 400 ml of sugar sweetened beverages per day compared to the control group. At 2 year follow-up, this difference was not statistically significant.

Jones et al. [9] examined the effect of an internet-facilitated intervention for weight maintenance and binge eating in adolescents. The control was a wait-list control group, and outcomes included BMI, binge eating behaviours, fat and sugar intake, depression and programme adherence. This study reported no statistically significant differences in outcomes between groups. The only within group statistically significant finding in the intervention group was a reduction in objective and subjective binge episodes from baseline assessment to post-treatment and follow-up assessment.

A further web-based RCT by Nawi and Jamaludin [26] attempted to determine the effectiveness of an internet-based intervention (obeseGO!) to address obesity among adolescents in Kuala Lumpur compared to a control group who were provided with written health education pamphlets. Outcomes measured were anthropometric measures; specifically, BMI, WC and % BF. This study found no statistically significant reduction in any outcomes between groups. However, on within-group analyses, mean BMI, WC and % BF in the obeseGO! group were statistically significantly lower after the intervention.

Finally, Williamson et al. [23] conducted a study to test the efficacy of an internet-based lifestyle behaviour modification programme for African-American girls over a 2 year period. The control group was a passive internet health education programme. Outcomes measured were anthropometric measures, weight loss behaviours and website use. At the 6 month follow-up, participants in the intervention group in comparison to the control group had statistically significantly reduced their % BF, however, this difference was not sustained and at the end of the 24 month intervention period, there was no difference in % BF between the two groups. Adolescents in both groups reported a statistically significant improvement in exercise and a reduction in overeating, in comparison with baseline.

To summarise, one study out of the five had a statistically mean difference for the intervention compared to the control for one of its weight-related outcomes; Chen et al. [27] found a small but statistically significant mean decrease in WHtR (0.01) compared to the control group with a *P*-value of 0.02. The remaining findings were not statistically significant when between group analyses were conducted and, as before, displayed a degree of heterogeneity in estimates in terms of trend of direction.

### Mobile phone communications

The third category of technology-based interventions, mobile phone communications, was explored in a single study by Nguyen et al. [29], where the effect of supplementary therapeutic contact as an additional support to a community-based weight-management programme for overweight and obese adolescents was examined (Table 4). Additional therapeutic contact took the form of telephone coaching, SMS or email communications. The control group was the weight-management programme alone. Outcomes measured were anthropometric measures, blood pressure, metabolic profile and self-reported psychosocial and lifestyle changes. Both groups demonstrated statistically significant reductions in BMI z-scores and WHtR as well as improvements in metabolic and psychosocial profiles. The study found that this technology-based intervention had no statistically significant impact on body weight, BMI, BMI z-score, WC and WHtR compared to the control group.

**Table 4:** Intervention and Control Characteristics, Outcomes, Follow-up Time Points and Key Findings for a Single Study Investigating the Effects of Mobile Phone-based Communications

Authors & year	Interventions	Intervention intensity & duration	Outcomes	Follow-up time points	Key findings	Weight-related outcomes	Mean difference	*P*-value*
Nguyen et al., 2013	1) Behavioural lifestyle programme plus: telephone/ SMS/email 2) Programme only	2 years During Phase 2 (22 months) adolescents received 14 telephone coaching sessions and 32 SMS +/ emails	1) Weight 2) BMI 3) BMI z-score 4) WC 5) WHtR 6) SBP & DBP 7) Metabolic profile 8) Self-reported psychosocial and lifestyle changes	2 months (end of Phase 1) 12 months 24 months (end of Phase 2)	- In both arms the programme produced statistically significant reductions in BMI z-score, WHtR and several metabolic and psychosocial improvements - Compared to the programme alone, the additional communication had no statistically significant impact on outcomes	Body weight (kg)	−2.1 (−7.14, 2.94)	0.42
						BMI (kg/m2)	−1.0 (−2.45, 0.45)	0.18
						BMI z-score	−1.0 (−0.24, 0.04)	0.18
						WC (cm)	−0.5 (−4.36, 3.36)	0.18
						WHtR	0.00 (−0.02, 0.02)	1

*Abbreviations* - *SMS* short message service, *BMI* body mass index, *WC* waist circumference, *WHtR* waist to height ratio, *SBP* systolic blood pressure, *DBP* diastolic blood pressure

*P-values in bold are statistically significant at 5% significance level

In terms of statistically significant findings, active video gaming was demonstrated to have had a positive impact on weight outcomes in 40% of studies (2/5). However, it is important to note that, within this review, five out of eleven studies focused on the use of active video games, while only one study explored mobile phone-based interventions, and this simple disparity in number of studies conducted to date may partly account for this outcome.

### Quality assessment

When methodological quality was evaluated [22], all studies fell into the category of poor quality. Figure S3 is a pictorial risk of bias summary for each of the studies (See Additional file 3). Such low quality, and challenges in judging the presence of selection, performance, attrition and other bias in the included studies renders the evidence less trustworthy and creates a challenge for making reliable recommendations for future practice. The high risk of performance bias found in three studies is concerning particularly in the lack of blinding of participants and personnel.

## Discussion

The aim of this systematic review was to assess the effectiveness of technology-based interventions, namely active video gaming, internet or web-based interventions and mobile phone communications, employed as secondary prevention, to address childhood obesity. However to combat obesity successfully, multicomponent interventions considering both diet and physical activity are likely to be required.

The review assessed the effect of these interventions on weight-related outcomes including weight, BMI percentile, BMI z-score, % BF, WC and WHR, compared to a control group, which had been assigned either nil intervention, a non-technology-based intervention or a similar technology-based intervention but with a more passive or sedentary element. The review allowed comparison between the three types of technology-based interventions by analysing the proportion of studies demonstrating positive effects on weight-related outcomes.

Of the eleven studies reviewed, three showed a positive relationship between technology-based interventions and weight-related outcomes in overweight or obese children. The findings of this review overall showed a weak evidence base regarding the role of technology-based interventions, employed as secondary prevention, to address childhood obesity.

Of the five studies, which explored the effect of active video gaming or exergaming, two demonstrated a statistically significant mean difference for the intervention compared to the control for all or some of the weight-related outcomes. Maddison et al. [30] found statistically significant beneficial results for BMI, BMI z-score, body weight and % BF when comparing the effects of active video games to sedentary video games. Trost et al. [16] compared a paediatric weight management programme in conjunction with active video gaming to the weight management programme alone and found a statistically significant mean difference for BMI z-score between and within groups.

In terms of studies which explored the effect of internet or web-based interventions, one out of five studies, which was by Chen et al. [27] found a small but statistically significant mean decrease in WHtR compared to the control group. There was also a statistically significant reduction of WHtR within the intervention group.

A single study by Nguyen et al. [29], which compared the effect of a behavioural lifestyle programme, supported by telephone and text messages or email, with the lifestyle programme alone found no statistically significant between group differences. However, within both groups, there were statistically significant reductions in BMI z-score and WHtR.

Active video games and internet-based interventions had some statistically significant effects on weight-related outcomes compared to control groups, while mobile phone communications had no effect. However, it is important to note that only one study explored mobile phone-based interventions. This disparity may partially explain the outcomes. While internet-based interventions were examined in a similar number of studies, they demonstrated a lower proportion of positive effects. The review suggests that active video games were most effective in terms of demonstrating positive effects on weight-related outcomes. However, in the absence of a direct comparison between the various types of intervention it is difficult to ascertain the efficacy of one over the other.

Statistically significant results were noted with interventions of longer duration for active video games. However, no association between longer duration and statistically significant positive outcomes was noted for internet or web-based interventions.

The findings of this systematic review produced results which were consistent with other, similar systematic reviews, of which there is a limited number. Previous systematic reviews have demonstrated the potential of technology-based interventions, such as internet-based weight management programmes, mobile phones and active video games, to educate and help overweight and obese children. An et al. [14] examined RCTs assessing the effect of web-based weight management programmes alone for children and adolescents. The review found that, in two out of three studies, where internet-based interventions were compared with a control group, the BMI in the internet-based intervention groups was statistically significantly reduced. A further systematic review by Chen et al. [32] confirmed the potential of technology-based interventions, similar to those assessed in this review, for reducing the risk of obesity in adolescents. The current systematic review addressed a specific gap in knowledge in that it focused on the role of various, current technology-based interventions, employed as secondary prevention as opposed to primary, to address childhood obesity.

Findings from this review, which demonstrate the potential of technology-based interventions in improving weight related outcomes in a population of overweight or obese children, are consistent with previous literature. This is the first review to search the literature to identify all studies, which explore the effectiveness of technology-based interventions, employed as secondary prevention, to address childhood obesity. Furthermore, this review identified active video games as potentially the most effective technology-based intervention in addressing obesity in children.

### Strengths and limitations

In terms of study strengths, the majority of studies used and described method of randomisation appropriately, leading to a reduced likelihood of selection bias. In addition, there were low levels of selective reporting, leading to a reduced likelihood of reporting bias. All weight-related outcomes in the included studies were measured objectively, reducing the likelihood of inaccuracy.

Study weakness included a lack of methodological clarity. This was partly due to the lack of blinding of participants, which would have proved difficult to carry out. All included studies proved to be of poor quality when AHRQ standards were applied, resulting in a potential loss of confidence in the study outcomes. The eleven studies were conducted by different investigators and different institutions in various locations worldwide; study variance must, therefore, be taken into consideration. Study participants varied in terms of gender, age, ethnicity and weight status. Studies differed in terms of interventions investigated, aims, follow-up times, attrition rates and outcomes measured. This heterogeneity among included studies restricts the ability to make direct comparisons and draw reliable conclusions.

Strengths of the review included clarity and robustness of its methods, which were reproducible and transparent. A methodical approach was used to determine the final eleven studies, allowing potential for repeatability. A comprehensive search strategy was employed and appropriate eligibility criteria applied to the potentially relevant studies. Reasons for exclusion were documented to ensure transparency. A second reviewer (RM) independently screened the full text articles to ensure consensus on study selection. Furthermore, the bibliographies of included articles were checked to identify other articles not listed in the two databases. The methodological quality of each study was considered when drawing conclusions from this review. Justification for not carrying out a meta-analysis was given, with an appropriate narrative approach adopted.

Two large, relevant databases were searched for the review and all reference lists were hand-searched. However, it may be that relevant studies were missed, which might impact on conclusions and recommendations. The search was limited to English language, which may introduce a language bias. Moreover, the initial screening of study titles and abstracts was conducted by one author only which in turn may be regarded as a limitation.

## Conclusions

The review suggests that technology-based interventions may be effective in terms of demonstrating positive effects on weight-related outcomes, and that the effectiveness may depend on the type of technology used. However, without a direct comparison between the various types of intervention, it is difficult to draw a firm conclusion. Moreover, it was difficult to identify common characteristics in papers with similar findings and, due to the heterogeneity and poor quality of included studies, caution should be used when interpreting the results in relation to the review question and when translating to professional practice.

The review highlights that there is some potential of technology-based interventions, but also the need for further investigation and careful evaluation. Future research needs to address the aforementioned limitations of currently published findings. With only one included study assessing the effectiveness of mobile phone communications, more studies should be initiated in this area. Further research might establish if the setting where the intervention takes place has an impact on the efficacy of the intervention. No guidelines currently exist regarding the optimal intervention duration period, and more research needs to be undertaken to determine this. Longer intervention duration periods and associated follow-up periods would allow for assessment of long-term effects and their sustainability. In the future, consideration should also be given to the significance of the age of participants as children and adolescents might differ in their attitudes and response to the various technology-based interventions.

## Supplementary information

**Additional file 1: Figure S1.** Search strategy used in Embase.

**Additional file 2: Figure S2.** Flow diagram summarising the study selection process.

**Additional file 3: Figure S3.** Risk of Bias Summary: Judgements on each risk of bias item for included.

## Data Availability

Data sharing is not applicable to this article as no datasets were generated or analysed during the current study.

## Abbreviations

RCTs: randomised controlled trials
BMI: body mass index
PRISMA: Preferred Reporting Items for Systematic Reviews and Meta-Analysis
BF: body fat
WC: waist circumference
WHR: waist to hip ratio
CDC: Centres for Disease Control and Prevention
IOFT: International Obesity Task Force
PA: physical activity
DBP: diastolic blood pressure
SMS: short message service
AHRQ: agency for healthcare research and quality
