# Supplementary figures and images for: A systematic review to assess the effectiveness of technology-based interventions to address obesity in children
Source: BMC Pediatr. 2020 May 22;20:242. doi: 10.1186/s12887-020-02081-1 (PMC7243328; doi:10.1186/s12887-020-02081-1)

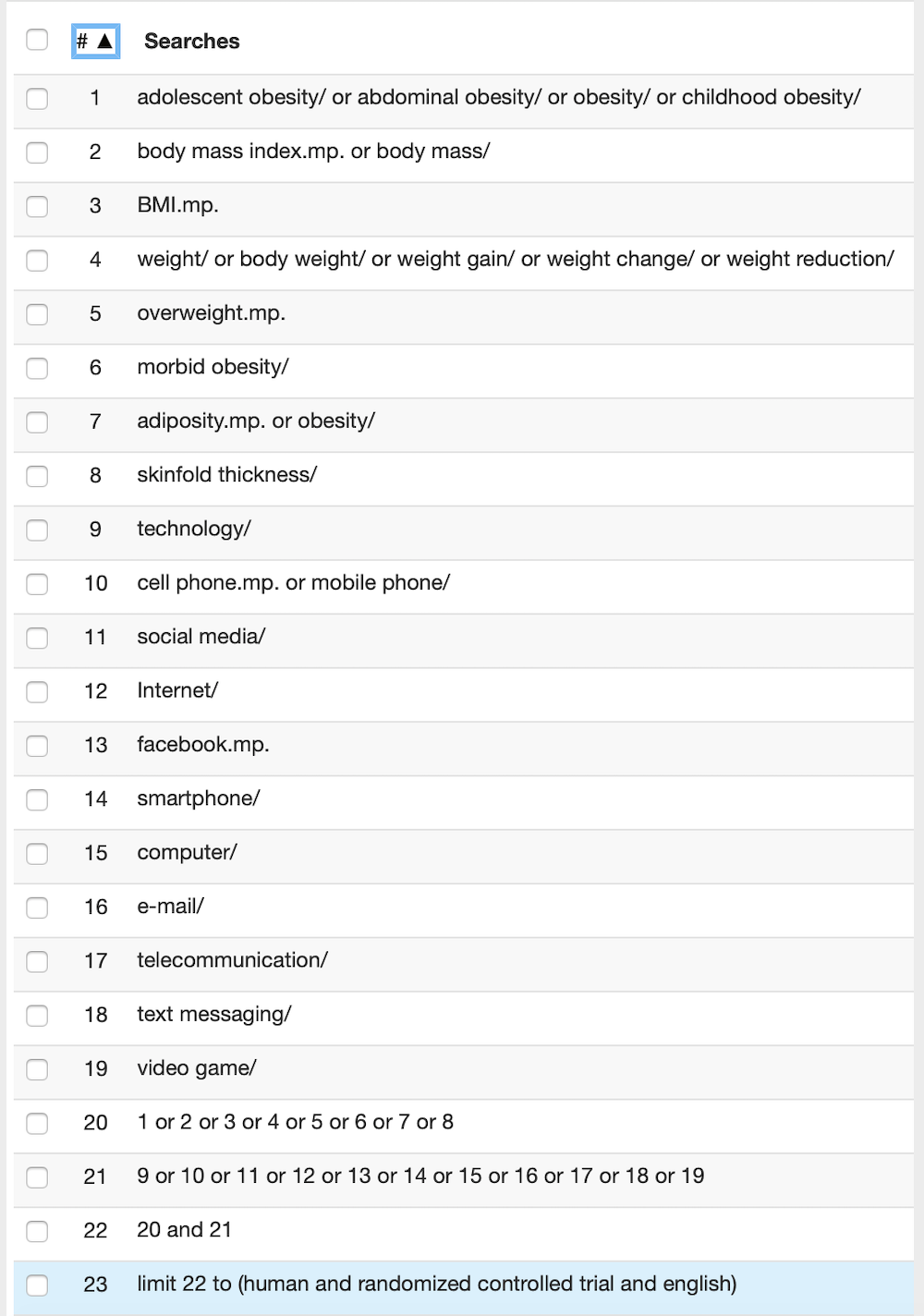

Supplement: Supplementary file 1 — Additional file 1: Figure S1. Search strategy used in Embase. [file 12887_2020_2081_MOESM1_ESM.docx]

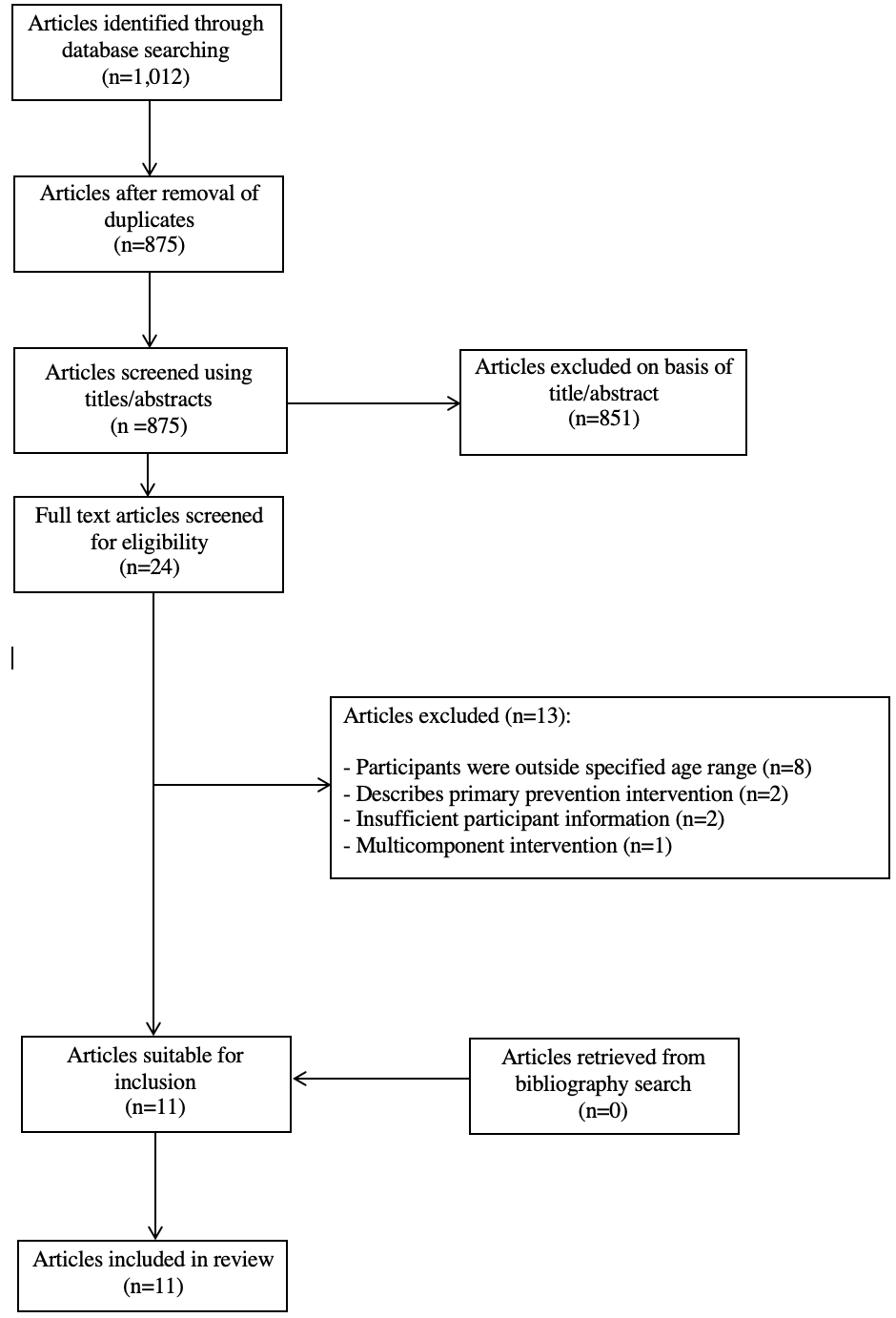

Supplement: Supplementary file 2 — Additional file 2: Figure S2. Flow diagram summarising the study selection process. [file 12887_2020_2081_MOESM2_ESM.docx]

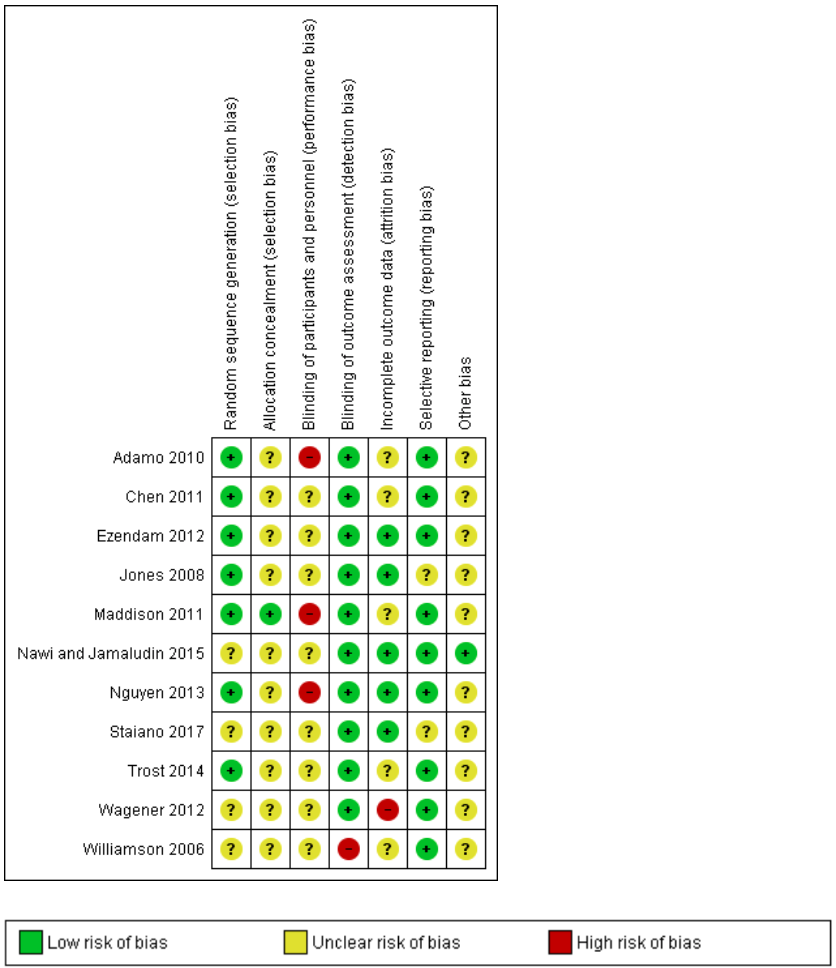

Supplement: Supplementary file 3 — Additional file 3: Figure S3. Risk of Bias Summary: Judgements on each risk of bias item for included. [file 12887_2020_2081_MOESM3_ESM.docx]
